# Clinical Characteristics and Outcomes of Patients Admitted in Cardiac Intensive Care Unit with Cardiogenic Shock: A Single-Center Study

**DOI:** 10.3390/diseases13090302

**Published:** 2025-09-13

**Authors:** Konstantinos C. Siaravas, Aidonis Rammos, Aris Bechlioulis, Christos D. Floros, Eftychia Papaioannou, Ioanna Samara, Ilektra Stamou, Petros Kalogeras, Spyridon Athanasios Sioros, Vasilis Bouratzis, Lampros Lakkas, Christos S. Katsouras, Katerina K. Naka, Lampros K. Michalis

**Affiliations:** 1 First Department of Cardiology, University Hospital of Ioannina, 45110 Ioannina, Greece; siaravaskon@gmail.com (K.C.S.); cskats@yahoo.com (C.S.K.); 2 Second Department of Cardiology, University Hospital of Ioannina, 45110 Ioannina, Greece; a.rammos@uoi.gr (A.R.); md02798@yahoo.gr (A.B.); christos.floros@yahoo.com (C.D.F.); efpapaioannou@hotmail.com (E.P.); pkalog90@yahoo.com (P.K.); spsior@yahoo.com (S.A.S.); v.bouratzis@gmail.com (V.B.);; 3 Service de Cardiologie, Hopitaux du Pays du Mont Blanc, 74700 Sallanches, France; ioan.samara31@gmail.com; 4 Department of Physiology, University of Ioannina, 45110 Ioannina, Greece; ftpcavalier52@gmail.com

**Keywords:** cardiogenic shock, acute myocardial infarction, heart failure, intra-aortic balloon pump, mechanical circulatory support, mortality

## Abstract

Background: Cardiogenic shock (CS) is a life threatening condition marked by inadequate tissue perfusion due to impaired cardiac output. Despite advances in diagnosis and management, mortality remains unacceptably high. Objective: This prospective, single-center observational study aimed to characterize the clinical profile, management strategies, and short- and long-term outcomes of patients with CS. Methods: Seventy patients (SCAI stages B–E) admitted to the Cardiac Intensive Care Unit (CICU) of a tertiary university hospital over a 24-month period were enrolled. Demographic, clinical, hemodynamic, echocardiographic, and biochemical data were collected. The primary outcomes were in-hospital, 1-month, and 1-year mortality. Secondary outcomes included the use of mechanical circulatory support (MCS), mechanical ventilation (MV), and continuous renal replacement therapy (CRRT). Results: Acute myocardial infarction-related CS (AMI-CS) and heart failure-related CS (HF-CS) accounted for 64% of cases. The overall in-hospital mortality was 49%. SCAI stage C was independently associated with higher mortality at all time points compared with stage B. Key predictors of mortality included higher SCAI stage, elevated lactate and creatinine levels, and reduced cardiac output. Intra-aortic balloon pump (IABP) use was more frequent in AMI-CS. Conclusions: CS continues to be associated with poor prognosis, particularly in patients with higher SCAI stages. Standardized clinical pathways and national registries are urgently needed to guide evidence-based and resource-appropriate care.

## 1. Introduction

Cardiogenic shock (CS) may be a final pathophysiological state for many cardiovascular disorders with potentially catastrophic consequences for patients [1]. Acute coronary syndromes (ACSs), chronic heart failure syndromes (HF) including ischemic cardiomyopathy, other cardiomyopathies (CMP), myocarditis as well as valvular heart disease (VHD), arrhythmias, post cardiac arrest syndrome (PCAS), and pulmonary embolism (PE) may all lead to CS during their course [2]. From a clinical perspective, the classical CS (SCAI C) is defined as a disorder of low cardiac output that results in sustained tissue hypoperfusion, irrespective of the underlying blood pressure [3], while SCAI B is defined as low cardiac output without tissue hypoperfusion. Despite efforts for a universal CS classification, there is great heterogeneity in terms of the etiologic, pathophysiological, and hemodynamic features among patients [4]. Regardless of the cause, the reduced cardiac output has detrimental effects in vital organ perfusion and function, thus rendering early recognition crucial in order to initiate etiopathophysiologic treatment and hemodynamic support aiming to reverse detrimental sequelae [5].

CS has a rising prevalence. Its mortality remains high despite a steady small decline in the adjusted trends of in-hospital mortality [1]. ACS has traditionally been the most common cause, and complicate around 5–10% of cases of myocardial infarction (MI) [6]. The current short-term mortality of CS related to ACS is around 40% [7] with the 1-year mortality approaching 50% [8], while over the previous decade, the 1-year mortality has been around 50–70%, with a high incidence of readmissions (18%) for those who survived the index event [9]. Another very common cause of CS is the acute decompensation of chronic HF syndromes, which presents with high variability and remains incompletely understood [10]. Currently, there is an ongoing research interest regarding the early diagnosis and categorization of the clinical syndrome with respect of its prognosis, the standardization of care for these patients, the organization of cardiac intensive care units (CICUs) in order to optimize CS management, and the proper implementation of MCS in clinical practice; it is expected that these advancements in knowledge will translate to a decreased mortality amongst CS patients over the following years [11].

The early clinical suspicion of CS is undoubtedly important, while physical examination and biochemical figures (e.g., levels of lactic acid) helping in prognostication are of utmost importance even before the application of advanced hemodynamic monitoring. Furthermore, distinction of (a) the shock phenotype (left ventricular, right ventricular, or biventricular shock), (b) the severity of the shock according to the Society of Cardiovascular Angiography Interventions (SCAI) staging, and (c) the recognition of the underlying responsible cause, are important for the management of these patients [12]. Many risk scores have been used for the prognostication of such patients. However, a lack of their universal acceptance and validation prevents their wide usage in clinical practice [13]. Many social, demographic, clinical, and logistic factors play an important role in the therapeutic decisions regarding patients with CS. Clinical questions arise regarding the use of MCS in these patients (i.e., timing of initiation, escalation and weaning, type of device according to the phenotype, SCAI class, and etiology of CS, etc.) [14].

The aim of the current prospective observational study was to primarily define the outcomes of the in-hospital, 1-month, and 1-year mortality of patients with CS that were admitted to the CICU of a tertiary university hospital, with emphasis on its etiology, phenotype, and SCAI classification stage, and the search for gender-specific differences. These real world data are scarce in Greece. In addition, the current study investigated secondary outcomes like the need for advanced support interventions with the use of MCS devices, continuous renal replacement therapy (CRRT), and invasive mechanical ventilation (MV) and aimed to compare treatment choices according to the available resources, to emphasize the need for national based treatment protocols and registries, and the conceptualization of shock teams and centers at a national level in order to assist in better treatment for CS patients.

## 2. Materials and Methods

### 2.1. Patient Selection

This study was a single-center, prospective, observational registry that enrolled 70 consecutive patients with CS with SCAI stages B to E. All patients were admitted to the CICU of the University Hospital of Ioannina, Greece, within a period of 24 months (November 2022 to November 2024). Patients that survived the initial hospitalization were followed in the HF clinic for 12 months after their discharge. No patients were lost during the follow-up period. The mortality rate (in-hospital, 1-month, and 1-year), the hospitalization duration, the need for MCS as well as the use of CRRT and of invasive MV during hospitalization were recorded.

The enrolled patients fulfilled the SCAI criteria from stages B to E. Stage B: clinical evidence of relative hypotension (systolic blood pressure, SBP, below 90 mmHg or mean arterial pressure, MAP, below 65 mmHg) or tachycardia (more than 100 beats per minute) without hypoperfusion (lactate < 2 mmol/L); stage C: hypoperfusion requiring more than volume resuscitation (use of inotropes, vasopressors, or mechanical support) to restore perfusion (lactate > 2 mmol/L); stage D: deterioration necessitating further escalation with multiple vasopressors or addition of MCS; and stage E: circulatory collapse, frequently in refractory cardiac arrest with ongoing cardiopulmonary resuscitation [15]. The only exclusion criteria were: age <18 years old, pregnancy, and refusal to give informed consent.

The following definitions were used regarding the etiology of CS: (a) an HF-CS term was used for patients with a decompensation of a known chronic HF syndrome, (b) an AMI-CS term was used for a diagnosis of acute heart failure syndrome with an ACS as the primary cause, (c) VHD was used for valvular disorders (moderate or severe valvular stenosis or regurgitation) that led, as a primary cause, to the acute HF episode or decompensation, (d) PCAS was used for patients that survived after an in- (IHCA) or out-of-hospital cardiac arrest (OHCA), and (e) the term arrhythmias was used only for bradyarrhythmias and hemodynamically stable ventricular tachycardia (episodes of hemodynamically unstable ventricular tachycardia and episodes of ventricular fibrillation were clustered as PCAS). The following shortcuts were used for counting units: bpm for beats per minute; mmHg for millimeters of mercury; mg/dl for milligrams per deciliter; mEq/L for milliequivalents per liter; IU/L for international units per liter; pg/mL for picograms per milliliter; mmol/L for millimols per liter; cm for centimeters; and mm for millimeters.

The registry was approved by the local hospital Scientific Board Committee (protocol code: 13203 and date of approval: 27 April 2022).

### 2.2. Patient Management

As soon as a patient was admitted to the CICU, the etiology of the CS was briefly determined (ACS vs. non ACS, mainly HF). A thorough physical examination, along with a focused echocardiogram, was performed, and a full blood count with a biochemical profile was ordered. Inotropic support was initiated if the SBP was < 90 mmHg or MAP was < 65mmHg. In case of AMI-CS, the patient was transferred immediately to the Cath-Lab, and coronary angiography and ad hoc PCI took place (the latter according to the current guidelines) [2]. IABP prior to intervention was inserted in complex PCI cases or in non-ACS cases when the hemodynamics were not improved after inotrope administration. A Swan–Ganz catheter was either inserted at the Cath-Lab after PCI or in the CICU for non-ACS patients. In the CICU, all patients were subjected to invasive monitoring and detailed echocardiographic evaluation.

Patients with worsening or not improving SCAI stage C despite inotropic support were managed using the following steps: (a) escalation of inotropic and vasopressor therapy, (b) insertion of IABP in patients without such a device, and (c) if further deterioration was noticed (with lactate > 2 mmol/L, creatinine double or 50% drop in glomerular filtration rate, increased liver function tests, SBP < 90 mmHg or MAP < 60 mmHg, and drugs or IABP unable to maintain BP above those targets) [15], the need for an advanced MCS was documented (Impella for LV and ECMO for RV and biventricular phenotypes). The reason for mere documentation and not the utilization of the above devices was their unavailability at both our institution as well as the vast majority of Greek NHS hospitals.

A full blood count with biochemical profile was carried out every 12 h for the first 48 h and then daily during their hospitalization.

Weaning from IABP was recommended based on the continuous monitoring of clinical parameters (urine output > 0.5 mL/kg/min, MAP > 65 mmHg), laboratory parameters (blood lactate < 2 mmol/L), and hemodynamic and echocardiographic indicators [Cardiac Power Output (CPO) > 0.6 W, Cardiac Index (CI) > 2.2 L/min/m^2^]. Continuous improvement of the above parameters rendered device removal feasible. Furthermore, discontinuation of support took place when irreversible complications and/or severe comorbidities were evident. Additional therapeutic decisions were made on a basis of a collaborative, multidisciplinary approach involving the whole healthcare team (interventional cardiologists, CICU cardiologists, HF experts, electrophysiologists, and cardiac surgeons) and either the patients (when mentally able) or their families.

For patients discharged alive from hospital, a follow-up was scheduled at 1-month and 1-year after the index event. All of the above data were recorded anonymously on the prospective registry case report form (CRF) with an individualized ID for each patient.

### 2.3. Statistical Analysis

Data were extracted directly from the CRF. All continuous variables were expressed as the mean value ± standard deviation and categorical variables as frequencies *n* (%). Missing data for continuous variables were managed with pairwise deletion (using only the available data without dropping the row completely) and categorical variables with the insertion of a new category of “unknown” value. Since outliers were accurate but rare values, they were managed with winsorizing (replacing the extreme values with maximum and minimum thresholds). The normality of the distribution for the independent continuous variables was tested with the Kolmogorov–Smirnov test and with statistical significance (*p*-value < 0.05); all variables followed a normal distribution.

Continuous variables were further analyzed with the student’s *t*-test for statistical significance. Categorical variables were tested for statistical significance with the chi-square test. For outcome measurements, odds ratios (ORs) were used, and statistical significance was tested with confidence intervals (CIs) and *p*-values. Univariable regression analysis was conducted to define possible predictors of mortality with binary logistic regression models.

Multivariable regression models were used for the adjustment for confounders but due to the small sample size, they did not yield statistical significant results. Thus, multivariable analysis was precluded due to the sample size limitations and event rates. The statistical software IMB SPSS Statistics 23 was used for the current analysis.

## 3. Results

### 3.1. Patient Characteristics

A total of 70 consecutive patients (*n* = 70) were enrolled during the two-year period of the study. Fifty-four (77%) were males, with a mean (±SD) age of 67 ± 24 years old. Forty-eight patients (68%) were categorized as SCAI stage C, twenty patients (28%) as stage B, one patient (2%) as stage D, and one patient (2%) as stage E. The underlying pathology was acute myocardial infarction (AMI-CS) in 24 patients (35%), decompensation of previously known HF (HF-CS) in 20 (29%), VHD in 11 (16%), arrhythmias in 6 (8%), PCAS in 5 (7%), tamponade in 2 (3%), acute myocarditis in 1 (1%), and PE in 1 patient (1%). According to the phenotype of CS, 47 patients (67%) had left ventricular (LV), 22 (31%) had biventricular (BiV), and 1 (2%) had right ventricular (RV) involvement. The demographic and all baseline characteristics of the patients are shown in Table 1. Supplementary Table S1 depicts the baseline characteristics on admission of patients with CS according to the SCAI classification stages, 1-month, and 1-year mortality.

The patients’ mean (±SD) heart rate was 96 beats per minute (bpm) (±38), the mean arterial systolic blood pressure was 108 mmHg (±36), the mean pulse pressure was 34 mmHg (±17), and the mean arterial pressure was 79 mmHg (±24 mmHg). Lactic acid levels in the arterial blood gas analysis were elevated in the majority of cases [mean (±SD): 3.6 mmol/dL (±3)]. Table 2 presents the vital signs and laboratory values on admission in the whole study population as well as in the subgroups of patients with AMI-CS and HF-CS. Supplementary Table S2 presents the vital signs, laboratory tests, echocardiographic, and hemodynamic measurements on the admission of patients with cardiogenic shock according to SCAI classification stages, 1-month, and 1-year mortality.

With regard to the management of our patients registered in the current database, all patients in the AMI-CS group underwent PCI, while two patients in the HF-CS group underwent revascularization (one with PCI and one with CABG). Fifteen patients (21%) needed an intra-aortic balloon pump (IABP), four (5.7%) needed the escalation of MCS (Impella: 3, ECMO: 1), sixteen (23%) MV support (due to acute respiratory failure), and seven (10%) CRRT (continuous veno-venous hemodiafiltration—CVVHDF; either due to acute kidney injury or for renal replacement as they were previously in intermittent renal dialysis).

Furthermore, 36 patients (51%) received noradrenalin, 16 (23%) dobutamine, 15 (21%) received double vasopressor treatment, 5 (7%) received triple, and 1 (1.4%) quadruple treatment.

**Figure 1:**
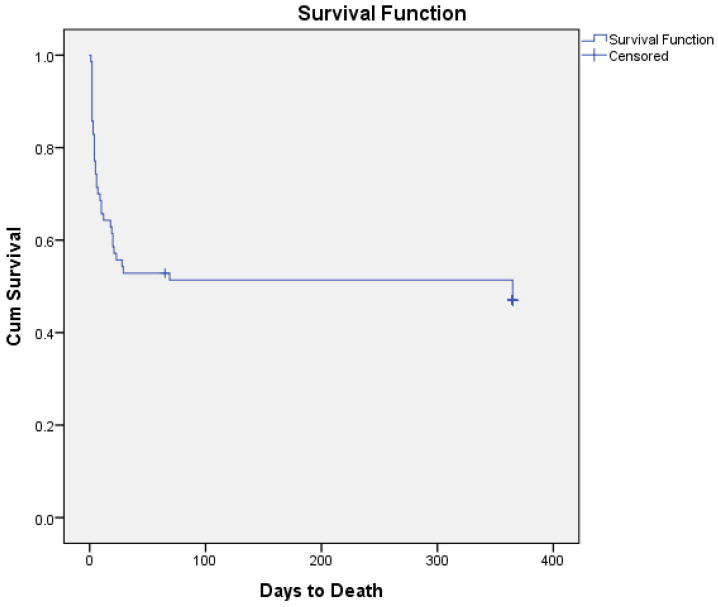
Kaplan–Meier curves of patient survival (in days) during the 1-year follow-up period.

Thirty-six patients (51%) out of the total population survived during the 1-month follow-up, and thirty-five patients out of fifty survived at the 1-year follow-up. All patients that survived during their hospitalization also survived the first month of the follow-up period. This translates to an in-hospital mortality rate of 49% in the current study. The causes of death were pump insufficiency leading to multiorgan failure in 18 patients (25.7%), and septic shock in 16 patients (23%). One patient (3%) out of the survivors developed non reversible brain damage. The mean hospitalization duration was 11 (±9) days for the total study population. Table 3 presents the outcomes of patients admitted with CS and with AMI-CS and HF-CS etiology. Figure 1 presents the Kaplan–Meier curves of patient survival (in days) during the 1-year follow-up period.

### 3.2. Subgroup Analysis on Baseline Characteristics

Although the studied population was small, most patients were admitted due to AMI-CS (35%) or HF-CS (29%). There were statistically significant differences among the AMI-CS and HF-CS patients in age (mean 64 vs. 72 years, *p* = 0.05), male sex prevalence (22 vs. 13 male, *p* = 0.02), history of CKD (6 vs. 11, *p* = 0.04), known CAD (5 vs. 10, *p* = 0.04), and known PH (0 vs. 4, *p* = 0.02). Furthermore, the AMI-CS subgroup had lower left ventricular ejection fraction (LVEF: 24% vs. 35%, *p* = 0.04), inferior vena cava diameter (IVC: 18 vs. 21 mm, *p* = 0.01), central venous pressure (CVP: 12 vs. 16 mmHg, *p* = 0.03), and Cardiac Index (CI: 1.7 vs. 2.1 L/min/m^2^, *p* = 0.001) as well as a higher high sensitivity troponin value (mean value 48,728 vs. 1632 pg/mL, *p* < 0.01) and the need for IABP support (11 vs. 4, *p* = 0.02). No statistically significant differences were noticed between AMI-CS and HF-CS with regard to MV support (6 vs. 3, *p*-value: 0.41), CVVHDF therapy (1 vs. 2, *p*-value: 0.44), and in-hospital mortality (10 vs.10, *p*-value: 0.58), respectively (Table 3).

Regarding the LVEF on admission, HF-CS patients had three distinct phenotypes; twelve (60%) had HF with reduced ejection fraction (HFrEF–LVEF ≤ 40%), three patients (15%) had mildly reduced ejection fraction (HFmrEF–LVEF ≥ 41–49%), and five patients (25%) had preserved ejection fraction (HFpEF–LVEF ≥ 50%). Respectively in the AMI-CS subgroup, only three patients (12.5%) had an LVEF ≥ 40%, while twenty-one patients (87.5%) had an LVEF < 40% on admission.

There was no statistically significant difference in SCAI stage on admission among the AMI-CS and HF-CS patients, while there was a tendency for a higher SCAI C stage in the AMI-CS patients (17 vs. 10, *p*-value: 0.17).

### 3.3. Subgroup Analysis on Outcomes

In the current study, patients were followed-up for 1-year after their initial admission to the CICU. The patients’ outcomes were tested for an association with CS phenotype (LV and BiV), SCAI stages B and C on admission, and with the most common etiologies of CS (AMI-CS and HF-CS). In the subgroups with AMI-CS and HF-CS, there were no significant differences in the in-hospital (OR: 0.71, CIs: 0.21–2.35, *p*-value: 0.58), 1-month (OR: 0.71, CIs: 0.21–2.35, *p*-value: 0.58), and 1-year mortality (OR: 0.58, CIs: 0.17–1.93, *p*-value: 0.37). In addition, there were no statistically significant differences in in-hospital and 1-month mortality between patients with the LV and BiV phenotypes (OR: 0.42, CIs: 0.14–1.20, *p*-value: 0.10), while the BiV phenotype was associated with a 21% increased risk of mortality (OR: 0.79, CIs: 0.64–0.89, *p*-value < 0.01) compared with the LV phenotype.

SCAI C stage on admission was associated with a higher mortality compared with SCAI B at any time point [in-hospital and 1-month: 17% increased risk of mortality (OR: 1.17, CIs: 1.05–1.61, *p*-value < 0.01), 1-year: 13% increased risk of mortality (OR: 1.13, CIs: 1.03–1.47, *p*-value < 0.01)]. In the current registry, patients with stage D (*n* = 1) and stage E (*n* = 1) class on admission did not survive hospitalization, while all patients discharged were alive in the first month and only one with HF-CS etiology (SCAI C on admission) and with the BiV phenotype died 2 months after the index event. This underlines the fact that in-hospital outcomes are possibly the most important for prognosis in CS patients. Table 4 summarizes the odds ratios for in-hospital, 1-month, and 1-year mortality among patients with AMI-CS and HF-CS, patients with the LV or BiV phenotype, and patients with stage B and C SCAI stage on admission.

Another correlation that was tested in the current analysis was the gender (biological classification)-related differences on outcomes and advanced support interventions. Out of the total database, 54 (77%) patients were males, and 16 (23%) females (*p*-value = 0.02). There were neither statistically significant differences on sex-specific in-hospital and 1-month mortality between groups (OR: 0.39, CIs: 0.12–1.28, *p*-value = 0.12), nor in 1-year mortality (OR: 0.42, CIs: 0.13–1.38, *p*-value = 0.15), while the values showed a trend for a higher ratio of adverse outcomes in women. On the other hand, there was a trend without reaching statistical significance for a male predominance in IABP usage (OR: 5.2, CIs: 0.63–23, *p*-value = 0.12) and in invasive MV support (OR: 5.7, CIs: 0.69–27, *p*-value = 0.10), while there was a female predominance in CRRT support (OR: 0.88, CIs: 0.85–9.11, *p*-value = 0.91). All of the above data highlight the need for sex-specific reports on the registries and studies of patients with CS.

### 3.4. Predictors of In-Hospital Mortality

Although the univariable analysis defined age, higher SCAI classification stage on admission, lower MBP, lower eGFR, higher lactic acid levels, and several echocardiographic and hemodynamic indices as possible predictors of in-hospital mortality, this was not depicted, perhaps due to the small sample size, when the multivariate analysis was used.

## 4. Discussion

In the current study, the most common etiology of CS was ACS (35%), with HF-CS being the second cause (29%). Although this is in accordance with previously reported data, the recent literature shows that HF has emerged as the primary cause (46%), possibly because other reasons for CS like CMPs and VHD have been included in that category [16]. Most patients in our study were males, both in the AMI-CS and HF-CS subgroups, in accordance with the literature. Patients with HF-CS were older compared with patients with AMI-CS [mean age ( ±SD): 72 (±26) vs. 64 (±26) years, *p*-value = 0.05] in accordance with the reported data [16,17] and had a significantly higher prevalence of previous CAD history (10 vs. 5 patients, *p*-value = 0.04).

In our study, 34 patients (49%) died during the index hospitalization, with significantly higher mortality in patients with SCAI stage C than B, at all of the time points examined [in-hospital, 1-month mortality (OR: 1.17, CIs: 1.05–1.61, *p*-value < 0.01) and 1-year mortality (OR: 1.13, CIs: 1.03–1.47, *p*-value < 0.01)]. Other studies have reported an in-hospital mortality between 12.4% and 36.4% for SCAI stage C patients, which is lower than that in our study (58%); however the 1-month and 1-year mortality resembled that of our patients (54.5% and 59% vs. 58% and 60%, respectively) [18,19]. In the current registry, 16 out of 34 (47%) deaths during hospitalization were due to sepsis, which is comparable to previously reported results [20]. The above data denote the importance of the early CS recognition and SCAI stage classification of each patient case, leading to a more appropriate treatment management. Furthermore, the early SCAI stage may serve as a pragmatic tool in settings with limited access to advanced MCS.

Patients with the AMI-CS phenotype were directly transferred to the Cath-Lab for coronary angiography, further revascularization, and possible MCS (IABP) insertion, while patients with HF-CS were initially stabilized pharmacologically, and later during hospitalization, they were supported with further MCS either during the time of coronary angiography or because of further hemodynamic compromise. The use of IABP as MCS in patients with HF-CS was denoted by the local availability sources and based on previously published data and a consensus statement reporting that in early SCAI class stages, IABP use in HF-CS may be useful for better hemodynamic support [12,21]. Regarding the pharmacological support with vasoactive drugs, there were no differences in the AMI-CS and HF-CS therapeutic strategy.

Four out of the fifteen patients in whom an IABP was inserted (5.7% of the total study population, 26.7% of the ones with IABP) needed escalation in MCS according to our patients’ management protocol (from three patients with AMI-CS: two needed Impella support and one needed VA-ECMO, while one patient with HF-CS needed Impella support). The outcome of these patients was as follows: one died due to sepsis, two died due to multiorgan failure, and one survived. Among the patients admitted with HF-CS, 25% was evaluated as in need of mechanical support, while recently published data have shown that 31% of chronic HF patients are managed with MCS devices (IABP, ECMO, or Impella) [22]. The number of patients included in the current registry was small, so we were unable to make certain conclusions on the importance of utilizing more advanced MCS than IABP, although it is possible that the two patients that died due to multiorgan failure could have survived if advanced MCS was available. However, taking into account that more advanced MCS devices are not available at both our institution and in the vast majority of Greek public hospitals, the current data can possibly be useful for health planning and budget allocation, at least in our health district.

Initial hemodynamic support in patients with CS is pharmaceutical and may require vasoactive medications and inotropes [23,24,25]. In the current study, noradrenalin and dobutamine were the main pharmacological treatment choices, either alone or in combination. The use of a combination of pharmacological therapies leads to a lower dosage of each drug and reduces the possible adverse effects and drug interactions [3]. Regarding the use of MCS during hospitalization, the IABP was implanted during the first 24-h in order to further reduce the dosage of vasoactive agent. Furthermore, better circulatory support and a reduction in the systematic responses of the CS pathophysiology can be achieved. In a recent study of patients with AMI-CS undergoing PCI, apart from MCS, 80% of patients received vasopressors, and in 40% of patients, two or more (up to four) agents were administered [26]. In our study, more than 50% of the patients received vasopressor drugs, with 23% of patients needing two and 8.5% of patients needing three or four different agents. Norepinephrine was the vasopressor more widely used (with dosages varying from 0.1 up to 0.7 mcg/kg/min), whereas for inotropic support, dobutamine was the one mostly administered (with dosages varying from 2.5 up to 15 mcg/kg/min) [27]. Moreover, four patients of the HF-CS subgroup (20%) also received levosimendan in an attempt to increase the inotropic effect [28].

There is an uncertainty with regard to the most appropriate uses of vasoactive drugs and how they affect mortality in these patients [29]. Limited data support the use of norepinephrine as the preferred first-line agent, and a retrospective analysis suggested similar outcomes with dobutamine and milrinone [30,31]. On the other hand, CS can be overlapped with vasoplegic syndrome in many cardiovascular conditions. Systemic inflammation in the phenotype of cardiogenic shock makes the administration of norepinephrine indicated for the correction of the pathophysiology of vasoplegic syndrome [32]. In another study, no specific drug revealed improved outcomes in patients with CS, though the evidence was of low quality [33]. A meta-analysis showed a significant reduced short-term mortality in patients with CS treated with levosimendan versus dobutamine (RR: 0.60, CI: 0.37–0.95), with a number needed to treat five patients with CS. Furthermore, the initial short-term benefit was not confirmed on a long-term basis [34].

Appropriate evidence-based decisions on MCS utilization are important for patients with CS, especially for those with AMI-CS and HF-CS. Pathophysiology differences among these two etiologies are important, resulting in higher mortality in the AMI-CS subgroup. The phenotype of ventricular involvement is crucial for the selection of an MCS device, while gender differences have statistical significance on survival [35]. In the current study, most patients with AMI-CS and HF-CS had the LV phenotype, with only a small number of patients (three vs. five, respectively) expressing the BiV phenotype. The DanGer Shock trial showed that patients with AMI-CS that were supported with a microaxial flow pump had a higher 6-month survival rate than those receiving the standard care [36]. In addition, the timing of MCS insertion in patients with AMI-CS has an impact on the overall survival. When implantation of the MCS was carried out prior to PCI, there was a statistically significant increase in the in-hospital, 30-day, and 6-month survival. From the different MCS devices that have been studied, only Impella and extracorporeal membrane oxygenator (ECMO) showed a reduced 30-day mortality, while only Impella had a reduced 6-month mortality [37].

In the current registry, only IABP was used, with AMI-CS patients having higher rates of IABP compared with those with HF-CS (11 vs. 4, *p*-value = 0.02). This can be partly explained by the structure of our management protocol, which suggests the use of IABP in HF-CS patients only with established CS, while it was more freely used in the AMI-CS subgroup (routine in complex PCI procedures). All patients had a pulmonary artery catheter (Swan–Ganz) inserted either at the Cath-Lab post PCI or at the CICU for the HF patients. Right heart catheterization is underused in clinical practice worldwide, despite the fact that it is associated with a higher survival in CS [38].

In the current registry, the only predictor of mortality was the SCAI stage on admission. Biochemical markers (e.g., glucose levels in non-diabetic patients) were not found to have an independent association with mortality rates, in disagreement with the current literature, possibly due to the small sample size [39]. It is possible that machine learning (ML) can further assist prognostic information and calculate the prognosis for patients with CS, leading thus to a more accurate risk stratification in these patients [40].

Future research needs to focus clinical trials on examining the role of different MCS devices on specific phenotypes of shock, SCAI stages, and etiologies. Additionally, the timing of the insertion, escalation, and weaning of support devices are important future perspectives. Moreover CS management should necessitate an appropriate organization of healthcare services to provide advanced therapies to properly selected patients in a timely manner, minimizing potential iatrogenic harm [30]. Since there are many technological advancements, the research on AI and ML could help with the systematic registration and therapeutic decision management of CS patients. Finally, optimization of the clinical trial design for research on CS will assist with more appropriate population selection, improve statistical analysis, and incorporate the patients’ perspective [41,42]. The configuration of national or regional shock teams and centers as well as AI-prognostication tools could bridge the current therapeutic gaps.

The limitation of the current study was its design as a single-center study, with a rather small number of patients, especially with non-AMI-CS, and higher SCAI stages (D and E). However, Greece lacks similar registries to portray the incidence of CS, its mortality rates, and the actual need for advanced MCS devices.

## 5. Conclusions

The management of CS remains an ongoing challenge. Despite the improvement in early diagnosis, the development of advanced therapies, and the organization of specialized CICUs, mortality remains high. More efforts are needed to identify the proper therapy for the right patient, taking into account the metabolic demands, the expertise as well as the local resources. Available MCS devices and novel pharmacological treatments may target the systemic CS syndrome, but early clinical suspicion, the use of selected biomarkers, and the treatment of comorbidities are of utmost importance. Finally, the current registry’s data can be utilized in guiding national protocols and policymaking until a national registry takes place. The data in such a registry, which will depict the actual prevalence and detect the real needs, will result in greater rational resource reallocation and effective treatment for this critically ill population.

## Figures and Tables

**Table 1:** Baseline characteristics on admission of patients with cardiogenic shock.

	Total * (*n* = 70)	AMI-CS * (*n* = 24)	HF-CS * (*n* = 20)	*p*-Values
**Gender: ** Male *n* (%):	54 (77)	22 (92)	13 (65)	**0.02**
**Age (years):**	67 (±24)	64 (±26)	72 (±26)	**0.05**
**Hypertension *n* (%):**	41 (58)	15 (63)	10 (50)	0.40
**Dyslipidemia *n* (%):**	46 (65)	14 (58)	11 (55)	0.82
**Diabetes mellitus *n* (%):**	29 (42)	10 (42)	10 (50)	0.58
**Current smoking *n* (%):**	26 (37)	11 (46)	8 (40)	0.69
**Chronic kidney disease *n* (%):**	25 (35)	6 (25)	11 (55)	**0.04**
**End stage renal disease *n* (%):**	4 (5)	1 (4)	2 (10)	0.44
**Previous history of CAD *n* (%):**	27 (38)	5 (21)	10 (50)	**0.04**
**Previous history of stroke *n* (%):**	2 (3)	2 (8)	0 (0)	**0.18**
**Previous history of pulmonary hypertension *n* (%):**	7 (10)	0 (0)	4 (20)	**0.02**
**SCAI class *n* (%): ** B: C: D: E:0	20 (28) 48 (68) 1 (2) 1 (2)	6 (25) 17 (71) 1 (4) 0 (0)	10 (50) 10 (50) 0 (0) 0 (0)	0.17
**Etiology of cardiogenic shock *n* (%): ** AMI-CS: HF-CS: Valvular: Arrhythmia: Post cardiac arrest syndrome: Tamponade: Myocarditis: Pulmonary embolism:	24 (35) 20 (29) 11 (16) 6 (8) 5 (7) 2 (3) 1 (1) 1 (1)	24 (100)	20 (100)	
**Phenotype *n* (%):** Left ventricular: Biventricular: Right ventricular:	47 (67) 22 (31) 1 (2)	21 (88) 3 (12) 0 (0)	15 (75) 5 (25) 0 (0)	0.28

AMI-CS: acute myocardial infarction-cardiogenic shock, CAD: coronary artery disease, HF-CS: heart failure-cardiogenic shock, SCAI: Society of Cardiovascular Angiography and Interventions, SD: standard deviation, * Continuous variables are expressed as the mean (±2 × SD), and nominal variables are expressed as the total number (frequencies).

**Table 2:** Vital signs, laboratory tests, echocardiographic, and hemodynamic measurements on the admission of patients with cardiogenic shock.

	Total * (*n* = 70) ±SD	AMI-CS * (*n* = 24) ±SD	HF-CS * (*n* = 20) ±SD	*p*-Values
**Heart rate (BPM):**	96 (±38)	97 (±36)	88 (±30)	0.10
**Systolic blood pressure (mmHg):**	108 (±36)	110 (±40)	112 (±42)	0.74
**Mean blood pressure (mmHg):**	79 (±24)	82 (±28)	78 (±22)	0.32
**Pulse pressure (mmHg):**	34 (±17)	42 (±14)	44 (±22)	0.41
**Glucose (mg/dL):**	213 (±116)	229 (±196)	209 (±186)	0.61
**Creatinine (mg/dL):**	1.9 (±1.4)	1.74 (±1.55)	2.04 (±1.21)	0.49
**Estimated GFR (ml/min/1.73m^2^):**	49 (±46)	53 (±52)	44 (±40)	0.07
**Potassium (mEq/L):**	4.3 (±1.48)	4.43 (±1.2)	4.35 (±1.4)	0.71
**Sodium (mEq/L):**	135 (±10)	134 (±10)	136 (±10)	0.30
**ALT (IU/L):**	149 (±80)	142 (±131)	238 (±206)	0.44
**AST (IU/L):**	196 (±93)	279 (±134)	116 (±94)	0.12
**High sensitivity troponin (pg/mL):**	18,000 (±12,000)	48,728 (±47,134)	1632 (±1601)	**0.01**
**Lactate levels (mmol/L):**	3.6 (±3)	3.9 (±3.6)	3.6 (±3.3)	0.55
**LVEF (%):**	30 (±24)	24 (±20)	35 (±26)	**0.04**
**TAPSE (mm):**	16 (±6)	18 (±6)	16 (±5)	0.15
**IVC (mm):**	21 (±10)	18 (±10)	21 (±8)	**0.01**
**LVOT VTI (cm):**	15 (±8)	24 (±8)	16 (±8)	0.17
**CVP (cmH2O):**	15 (±12)	12 (±10)	16 (±14)	**0.03**
**CO (L/min):**	3.8 (±2.6)	3.4 (±1.6)	3.9 (±1.9)	0.19
**CI (L/min/m^2^):**	2 (±1.4)	1.7 (±0.8)	2.1 (±0.8)	**0.01**
**CPO (Watts):**	0.72 (±0.56)	0.6 (±0.3)	0.6 (±0.4)	0.68
**PAPi:**	1.74 (±1.5)	1.7 (±0.8)	2.1 (±1.3)	0.56

ALT: aspartate aminotransferase, AMI-CS: acute myocardial infarction-cardiogenic shock, BPM: beats per minute, CI: Cardiac Index, CO: cardiac output, CPO: cardiac power output, CVP: central venous pressure, GFR: glomerular filtration rate, HF-CS: heart failure-cardiogenic shock, IVC: inferior vena cava, LVEF: left ventricular ejection fraction, LVOT VTI: left ventricular outflow tract velocity time integral, PAPi: Pulmonary Artery Pulsatility Index, SD: standard deviation, TAPSE: tricuspid annular plane systolic excursion. * Continuous variables are expressed as the mean (±2 × SD).

**Table 3:** Advanced support interventions and in-hospital outcomes of patients admitted with cardiogenic shock.

	Total * (*n* = 70)	AMI-CS * (*n* = 24)	HF-CS * (*n* = 20)	*p*-Values
**IABP:**	15 (21)	11 (45)	4 (20)	0.02
**Mechanical Ventilation:**	16 (23)	6 (25)	3 (15)	0.41
**CRRT:**	7 (10)	1 (4)	2 (10)	0.44
**Hospitalization survival:**	36 (51)	14 (58)	10 (50)	0.58
**In-hospital mortality:**	34 (49)	10 (42)	10 (50)	0.58
**Cause of death: ** Pump failure: Septic shock:	18 (25.7) 16 (23)	6 (25) 4 (16)	7 (35) 3 (15)	0.78
**Hospitalization duration:**	11 (±9)	11 (±10)	8 (±7)	0.11

AMI-CS: acute myocardial infarction-cardiogenic shock, CRRT: continuous renal replacement therapy, HF-CS: heart failure-cardiogenic shock, IABP: intra-aortic balloon pump. * Continuous variables are expressed as the mean (±2 × SD), and nominal variables are expressed as the total number (percentages).

**Table 4:** In-hospital, 1-month, and 1-year mortality for the total population and for subgroups of patients with AMI-CS and HF-CS, patients with the LV and BiV phenotype, and SCAI classes B and C on admission.

	AMI-CS *n* = 24 (%)	HF-CS *n* = 20 (%)	Odds Ratio	CIs	*p*-Values
**In-hospital:**	10 (41,7)	10 (50)	0.71	0.21–2.35	0.58
**1-month mortality:**	10 (41.7)	10 (50)	0.71	0.21–2.35	0.58
**1-year mortality:**	10 (41.7)	11 (55)	0.58	0.17–1.93	0.37
	**LV** ***n* = 47 (%)**	**BiV** ***n* = 22 (%)**	**Odds Ratio**	**CIs**	***p*-Values**
**In-hospital:**	20 (43)	14 (63.6)	0.42	0.14–1.20	0.10
**1-month mortality:**	20 (43)	14 (63.6)	0.42	0.14–1.20	0.10
**1-year mortality:**	20 (43)	15 (68.2)	0.79	0.64–0.89	**<0.01**
	**SCAI C** ***n* = 48 (%)**	**SCAI B** ***n* = 20 (%)**	**Odds Ratio**	**CIs**	***p*-Values**
**In-hospital:**	28 (58)	4 (20)	1.17	1.05–1.61	**<0.01**
**1-month mortality:**	28 (58)	4 (20)	1.17	1.05–1.61	**<0.01**
**1-year mortality:**	29 (60)	4 (20)	1.13	1.03–1.47	**<0.01**

AMI-CS: acute myocardial infarction-cardiogenic shock, BiV: biventricular, CIs: confidence intervals, HF-CS: heart failure-cardiogenic shock, LV: left ventricular, SCAI: Society of Cardiovascular Angiography and Interventions.

## Data Availability

The authors declare that anonymized data can be made available upon reasonable request, in line with open science principles.

## Supplementary Materials

The following supporting information can be downloaded at: https://www.mdpi.com/article/10.3390/diseases13090302/s1, Table S1: Baseline characteristics on admission of patients with cardiogenic shock according to SCAI classification stages, 1-month and 1-year mortality. Table S2: Vital signs, laboratory tests, echocardiographic and hemodynamic measurements on admission of patients with cardiogenic shock according to SCAI classification stages, 1-month and 1-year mortality.

## Abbreviations

The following abbreviations are used in this manuscript:

ACSs	Acute coronary syndromes
AI	Artificial intelligence
AMI	Acute myocardial infarction
AMI-CS	Acute myocardial infarction-related cardiogenic shock
APACHE-II	Acute Physiology and Chronic Health Evaluation II
BiV	Biventricular
Bpm	Beats per minute
CAD	Coronary artery disease
Cath-Lab	Catheterization laboratory
CICU	Cardiac intensive care unit
CI	Cardiac Index
CIs	Confidence intervals
CKD	Chronic kidney disease
CMP	Cardiomyopathies
CO	Cardiac output
CRF	Case report form
CRRT	Continuous renal replacement therapy
CS	Cardiogenic shock
CSP	Cardiogenic Shock Prognosis (score)
CVP	Central venous pressure
CVVHDF	Continuous veno-venous hemodiafiltration
DM	Diabetes mellitus
eGFR	Estimated glomerular filtration rate
ECMO	Extracorporeal membrane oxygenation
ESRD	End stage renal disease
GRACE	Global Registry of Acute Coronary Events (score)
HF	Heart failure
HF-CS	Heart failure-related cardiogenic shock
HFmrEF	Heart failure with mildly reduced ejection fraction
HFpEF	Heart failure with preserved ejection fraction
HFrEF	Heart failure with reduced ejection fraction
IABP	Intra-aortic balloon pump
ICU	Intensive care unit
ID	Identification
IHCA	In-hospital cardiac arrest
IVC	Inferior vena cava
LVEF	Left ventricular ejection fraction
LV	Left ventricular
LVOT VTI	Left ventricular outflow tract velocity time integral
MBP	Mean blood pressure
ML	Machine learning
MI	Myocardial infarction
MCS	Mechanical circulatory support
mPAP	Mean pulmonary artery pressure
MV	Mechanical ventilation
OHCA	Out-of-hospital cardiac arrest
OR	Odds ratio
PCAS	Post cardiac arrest syndrome
PCI	Percutaneous coronary intervention
PE	Pulmonary embolism
PH	Pulmonary hypertension
RV	Right ventricular
SAPS-II	Simplified Acute Physiology Score II
SCAI	Society of Cardiovascular Angiography and Interventions
SOFA	Sequential Organ Failure Assessment
SPAP	Systolic pulmonary artery pressure
STEMI	ST-segment elevation myocardial infarction
TAPSE	Tricuspid annular plane systolic excursion
TIMI	Thrombolysis in Myocardial Infarction (score)
VHD	Valvular heart disease
