# Supplementary material for: Clinical Characteristics and Outcomes of Patients Admitted in Cardiac Intensive Care Unit with Cardiogenic Shock: A Single-Center Study
Source: Diseases. 2025 Sep 13;13(9):302. doi: 10.3390/diseases13090302 (PMC12468640; doi:10.3390/diseases13090302)
Supplement: Supplementary file 1 [file diseases-13-00302-s001.zip › diseases-3775943-supplementary.pdf]

diseases

MDPI

Article-Supplementary Material

Clinical Characteristics and Outcomes of Patients Admitted in Cardiac Intensive Care Unit with Cardiogenic Shock. A single-Center Study

Konstantinos C. Siaravas[1], Aidonis Rammos[2], Aris Bechlioulis[2], Christos D. Floros[2], Eftychia Papaioannou[2], Ioanna Samara[3], Ilektra Stamou[2], Petros Kalogeras[2], Spyridon Athanasios Sioros[2], Vasilis Bouratzis[2], Lampros Lakkas[4], Christos S. Katsouras[1], Katerina K. Naka[2,] *and Lampros K. Michalis[2]

[1] First Department of Cardiology, University Hospital of Ioannina, Ioannina, Greece; siaravaskon@gmail.com (K.C.S.), cskats@yahoo.com (C.S.K.)
[2] Second Department of Cardiology, University Hospital of Ioannina, Ioannina, Greece; a.rammos@uoi.gr (A.R.), md02798@yahoo.gr (A.B.), christos.floros@yahoo.com (C.D.F.), efpapaioannou@hotmail.com (E.F.), ilektst@gamil.com (I.S.), pkalog90@yahoo.com (P.K.), spsior@yahoo.com (S.A.S.), v.bouratzis@gmail.com (V.B.), anaka@uoi.gr (K.K.N.), lamprosmihalis@gmail.com (L.K.M.)
[3] Service de cardiologie, Hopitaux du Pays du Mont Blanc, Sallanches, France; ioan.samara31@gmail.com (I.S.).
[4] Department of Physiology, University of Ioannina, Ioannina, Greece; LL: ftpcavalier52@gmail.com
* Correspondence: anaka@uoi.gr (K.K.N.)

Supplementary table S1. Baseline characteristics on admission of patients with cardiogenic shock according to SCAI classification stages, 1-month and 1-year mortality.

	1-month mortality* (n=34)	p-values	1-year mortality* (n=35)	p-values	Stage B* (n = 20)	Stage C* (n = 48)	p-values
**Gender:**							
Male n(%):	23 (67)	0.08	24 (69)	0.11	14 (70)	38 (79)	0.41
**Age (years):**	72 (±12)	0.06	37 (±12)	**0.008**	68 (± 28)	68 (± 24)	0.84
**Hypertension n(%):**	20 (58)	1.00	21 (60)	0.83	10 (50)	30 (62)	0.34
**Dyslipidemia n(%):**	22 (64)	0.79	22 (63)	0.55	12 (60)	33 (69)	0.48
**Diabetes Mellitus n(%):**	12 (35)	0.22	13 (37)	0.34	9 (45)	20 (42)	0.80
**Current Smoking n(%):**	8 (23)	**0.04**	9 (26)	0.08	8 (40)	16 (33)	0.60
**Chronic Kidney Disease n(%):**	16 (47)	0.07	16 (46)	0.11	7 (35)	18 (38)	0.84
**End Stage Renal Disease n(%):**	1 (2)	0.30	1 (3)	0.27	2 (10)	2 (4)	0.35
**Previous History of CAD n(%):**	15 (44)	0.31	15 (43)	0.41	6 (30)	20 (42)	0.36
**Previous History of Stroke n(%):**	2 (5)	0.15	2 (6)	0.16	0 (0)	2 (4)	0.35
**Previous History of Pulmonary Hypertension n(%):**	7 (20)	**0.05**	7 (20)	0.07	1 (5)	6 (12)	0.35
**Etiology of Cardiogenic Shock n(%):**							
AMI-CS:	8 (23)	0.41	9 (26)	0.53	6 (30)	17 (35)	0.26
HF-CS:	11 (32)		11 (31)		10 (50)	10 (21)	
Valvular:	7 (20)		7 (20)		2 (10)	9 (19)	
Arrhythmia:	2 (5)		2 (6)		2 (10)	3 (6)	
Post Cardiac Arrest Syndrome:	4 (11)		4 (11)		0 (0)	5 (10)	

Tamponade:	1 (2)		1 (3)		0 (0)	2 (4)	
Myocarditis:	1 (2)		1 (3)		0 (0)	1 (2)	
Pulmonary Embolism:	0 (0)		0 (0)		0 (0)	1 (2)	
**Phenotype n(%):**							
Left Ventricular:	18 (67)	0.11	19 (54)	**0.01**	16 (80)	30 (62)	0.34
Biventricular:	16 (31)		16 (46)		4 (20)	17 (36)	
Right Ventricular:	0 (0)		0 (0)		0 (0)	1 (2)	

AMI-CS: Acute Myocardial Infarction - Cardiogenic Shock, CAD: Coronary Artery Disease, HF-CS: Heart Failure - Cardiogenic Shock, SCAI: Society of Cardiovascular Angiography Interventions, SD: Standard Deviation, *Continuous variables are expressed as a mean (± SD) and nominal variables are expressed as total number (frequencies).

Supplementary table S2. Vital signs, laboratory tests, echocardiographic and hemodynamic measurements on admission of patients with cardiogenic shock according to SCAI classification stages, 1-month and 1-year mortality.

	**1-month mortality* (n=34) ±SD**	**p-values**	**1-year mortality* (n=35) ±SD**	**p-values**	**Stage B* (n = 20) ±SD**	**Stage C* (n = 48) ±SD**	**p-values**
**Heart Rate (bpm):**	98 (± 17)	0.17	98 (± 17)	0.22	91 (± 20)	97 (± 17)	0.19
**Systolic Blood Pressure (mmHg):**	106 (± 20)	0.23	106 (± 19)	0.21	117 (± 20)	106 (± 17)	**0.02**
**Mean Blood Pressure (mmHg):**	76 (± 13)	**0.03**	76 (± 13)	**0.03**	85 (± 12)	76 (± 11)	**0.006**
**Glucose (mg/dL):**	216 (± 104)	0.82	231 (± 114)	0.20	200 (± 109)	218 (± 122)	0.56
**Creatinine (mg/dL):**	2.3 (± 1.6)	**0.01**	2.3 (± 1.5)	**0.02**	1.63 (± 0.86)	2.12 (± 1.55)	0.19
**Estimated GFR (ml/min/1.73m²):**	35 (± 20)	**0.02**	36 (± 20)	**0.01**	55 (± 31)	44 (± 24)	0.12
**Potassium (mEq/L):**	4.49 (± 0.85)	0.08	4.4 (± 0.85)	0.14	4.15 (± 0.64)	4.41 (± 0.75)	0.18
**Sodium (mEq/L):**	134 (± 5.9)	0.24	134 (± 6.3)	0.06	136 (± 3.7)	134 (± 5.9)	0.19
**ALT (IU/L):**	237 (± 78)	**0.02**	232 (± 76)	**0.03**	54 (± 41)	189 (± 96)	0.12
**AST (IU/L):**	157 (± 38)	0.41	154 (± 37)	0.35	154 (± 73)	211 (± 55)	0.56
**High sensitivity troponin (pg/mL):**	14551 (± 6671)	0.55	14878 (± 6485)	0.57	16499 (± 3279)	19204 (± 7286)	0.84
**Lactate levels (mmol/L):**	4.4 (± 0.63)	**0.03**	4.4 (± 0.61)	**0.02**	1.8 (± 1.1)	4.3 (± 3.5)	**0.02**
**LVEF (%):**	30 (± 15)	0.86	30 (± 15)	0.86	30 (± 12)	30 (± 15)	0.97
**TAPSE (mm):**	18 (± 5)	0.13	18 (± 5)	0.12	20 (± 4.1)	21 (± 4.6)	0.35
**IVC (mm):**	22 (± 6)	0.11	21 (± 6.2)	0.26	21 (± 4.3)	20 (± 6.1)	0.67
**LVOT VTI (cm):**	14 (± 4)	**0.05**	14 (± 4)	**0.05**	17 (± 4.6)	15 (± 3.9)	0.14
**CVP (cmH2O):**	16 (± 6.6)	0.24	16 (± 6.6)	0.33	16 (± 7)	15 (± 7)	0.73
**CO (L/min):**	3.9 (± 1.6)	0.49	4 (± 1.6)	0.49	3.8 (± 0.85)	3.9 (± 1.5)	0.77
**CI (L/min/m²):**	2.05 (± 0.95)	0.64	2.05 (± 0.95)	0.64	2.1 (± 0.4)	1.9 (± 0.86)	0.67
**CPO (Watts):**	0.74 (± 0.36)	0.46	0.74 (± 0.36)	0.46	0.73 (± 0.15)	0.7 (± 0.34)	0.71
**PAPi:**	1.9 (± 0.39)	0.25	1.9 (± 0.39)	0.25	1.9 (± 0.51)	1.6 (± 0.18)	0.48

ALT: Aspartate Aminotransferase, AMI-CS: Acute Myocardial Infarction - Cardiogenic Shock, BPM: Beats per minute, CI: Cardiac Index, CO: Cardiac Output, CPO: Cardiac Power Output, CVP: Central Venous Pressure, GFR: Glomerular Filtration Rate, HF-CS: Heart Failure - Cardiogenic Shock, IVC: Inferior Vena Cava, LVEF: Left Ventricular Ejection Fraction, LVOT VTI: Left Ventricular Outflow Tract Velocity Time Integral, PAPi: Pulmonary Artery Pulsatility Index, SD: Standard Deviation, TAPSE: Tricuspid Annular Plane Systolic Excursion. *Continuous variables are expressed as a mean (± SD).
